# Optimizing Retrieval of Biospecimens Using the Curated Cancer Clinical Outcomes Database (C3OD)

**DOI:** 10.1177/1176935119886831

**Published:** 2019-11-18

**Authors:** Dinesh Pal Mudaranthakam, Elena Shergina, Michele Park, Jeffrey Thompson, David Streeter, Jinxiang Hu, Jo Wick, Byron Gajewski, Devin C Koestler, Andrew K Godwin, Roy A Jensen, Matthew S Mayo

**Affiliations:** 1 Department of Biostatistics & Data Science, University of Kansas Medical Center, Kansas City, KS, USA; 2 University of Kansas Cancer Center, Kansas City, KS, USA

**Keywords:** Informatics, data warehouse, biospecimen, clinical annotation

## Abstract

To fully support their role in translational and personalized medicine, biorepositories and biobanks must continue to advance the annotation of their biospecimens with robust clinical and laboratory data. Translational research and personalized medicine require well-documented and up-to-date information, but the infrastructure used to support biorepositories and biobanks can easily be out of sync with the host institution. To assist researchers and provide them with accurate pathological, epidemiological, and bio-molecular data, the Biospecimen Repository Core Facility (BRCF) at the University of Kansas Medical Center (KUMC) merges data from medical records, the tumor registry, and pathology reports using the Curated Cancer Clinical Outcomes Database (C3OD). In this report, we describe the utilization of C3OD to optimally retrieve and dispense biospecimen samples using these 3 data sources and demonstrate how C3OD greatly increases the efficiency of obtaining biospecimen samples for the researchers.

## Introduction

Robust biorepositories with readily available, high-quality, and well-annotated biospecimens play an essential role in advancing cancer research and personalized medicine.^1,2^ According to the biological material tracking recommendations of the International Society for Biological and Environmental Repositories (ISBER) and National Cancer Institute (NCI) Best Practices, biorepository information systems are expected to handle specimen tracking and have full query capability across all data stored. Both emphasize that biorepository information systems should consolidate data from different local systems within the institution (electronic medical records, cancer registries, pathology systems, etc)^3,4^ Biorepositories use databases that have 2 main components: an inventory system with compiled clinical information from different data sources and a link to samples in the physical repository. The inventory system enables administrators to retrieve on demand any requested clinical and diagnostic data on specific patients.^5-8^

Biospecimen Repository Core Facility (BRCF) plays a vital role at University of Kansas Medical Center (KUMC). Its ethical collection, storage, annotation, and distribution of high-quality biospecimens are essential to support translational research and investigator-initiated studies.

The BRCF was established in 1993 as breast tissue and serum repository. In 2011, changes were implemented to store bodily fluids (whole blood, urine, and saliva) with matching tumor. In addition, the BRCF launched the Early Detection Screening Project (EDSP) in 2013. This project is focused on collecting blood samples from women undergoing mammography screening and tracks changes in their medical history. The number of participants registered with the BRCF has grown by an average of 38% each year. In 2017, over 3700 participants consented to specimen collection and the BRCF collected solid tissue and blood samples at over 5800 patient visits.

**Figure 1.**
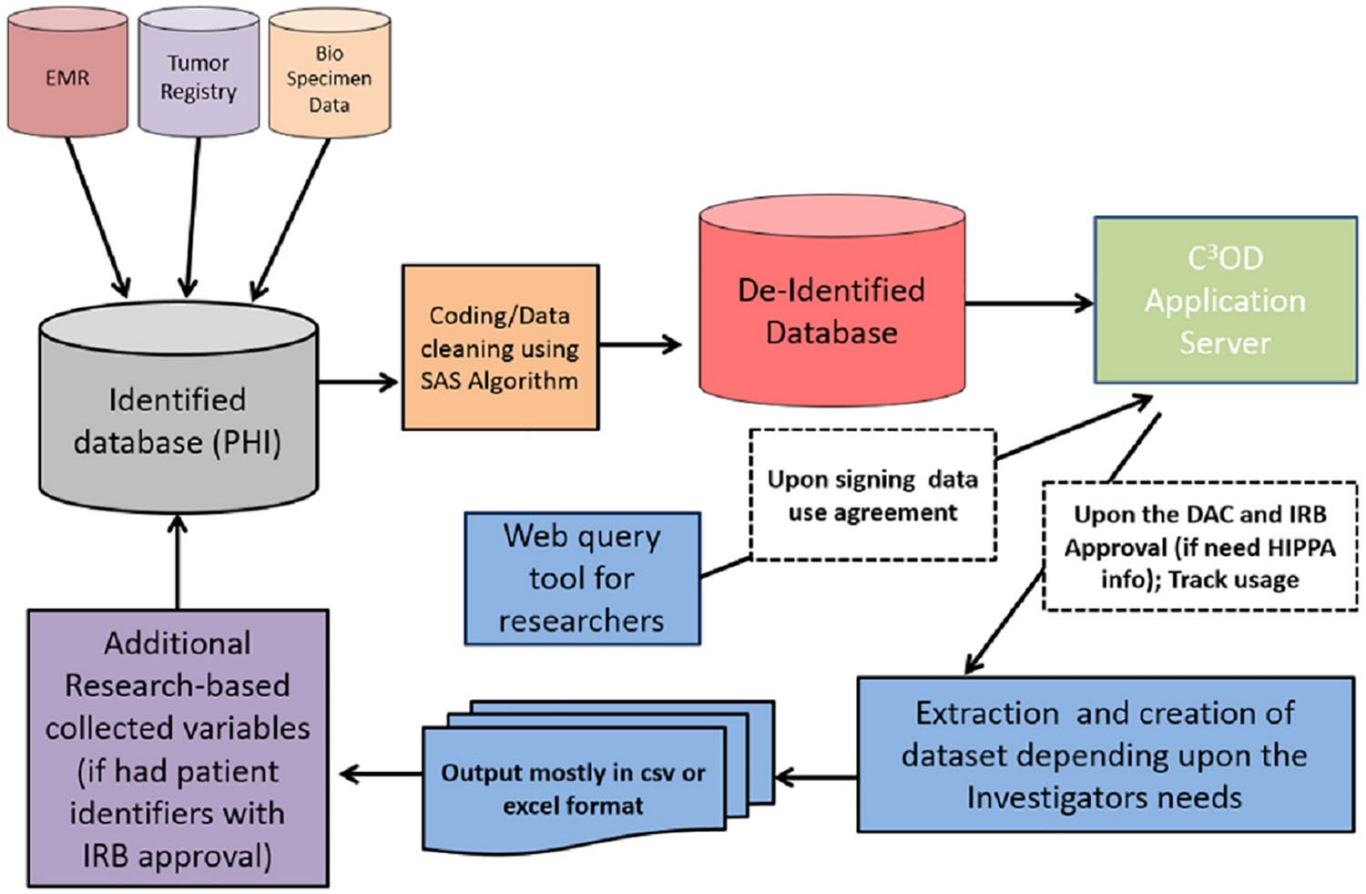
C3OD architecture. C3OD indicates Curated Cancer Clinical Outcomes Database; DAC, Data Access Committee; IRB, institutional review board; EMR, electronic medical record; HIPAA, health insurance portability and accountability act; PHI, protected health information; SAS, SAS Institute – Developer of Analytical software.

In 2017, C3OD was launched to address the lack of uniformity in clinical data collection and to create a unified system for the clinical annotation of the increasing number of specimens available to KUCC scientists for cancer research. C3OD merges data from the electronic medical record, the tumor registry, the biospecimen database, and the data registry to allow queries within a unified platform.^9^ The C3OD has 2 databases, identified and de-identified databases, to protect protected health information. This database extracts data from tumor registry with “NAACCR 16C” (tumor anatomic site, histology, etc), EMR (demographics, family history, diagnosis, comorbidities, etc), and biospecimen inventory information (sample type, annotation, the location of the sample within the freezer, etc). The 3 databases are populated in 3 stages: the extraction stage creates user-friendly querying (eg, gender is recorded into f/m/unknown from original 1/2/3), the transformation stage, and finally, the loading stage. The loaded data is also validated by the informatics administrator. This preparation and cleaning process takes about 2 weeks as the process is time-consuming. To be cost effective, the data refresh occurs once a month. Architecture is described in Figure 1: initial C3OD database architecture has been enhanced with biospecimen data being merged using the patient medical record number. The BRCF utilizes an inventory management system called OpenSpecimen developed by Krishagni (https://www.openspecimen.org/). Within the OpenSpecimen system, high-level patient information is being captured (medical record number, date of birth, patient name, gender) along with detailed information of the collection visit (sample type, sample class, quantity, collected by, collection date/time, pathology status, anatomic site, laterality, storage location). Variables used from tumor registry and biospecimen repository to populate C3OD are found in the Supplementary File 1. In this short report, we introduce the process and results of the BRCF operations utilizing C3OD, provide demonstrations of several examples, and discuss future directions for the BRCF.

## Materials and Methods

C3OD facilitates translational cancer research. Once a researcher obtains IRB and Data Access Committee (DAC) approvals, they can obtain patients’ protected health information. There are 3 major components involved in this process: the Kansas Cancer Registry houses data pertaining to cancer incidence information in the State of Kansas; the biospecimen repository (BRCF) collects, stores, and manages specimens of the cancer patients; and the Biostatistics and Informatics Shared Resource (BISR) provides statistical, data science, and informatics support to all cancer researchers. Each component nominates a representative to the DAC. The BRCF follows best practices in biospecimen storage and data management. Standard operating procedures, covering topics such as patient consent, specimen storage, and data management, have been developed to govern these processes. The BRCF preserves fresh and frozen tissue samples (both tumor and adjacent normal) stored at −190°C in liquid nitrogen vapor phase freezers, along with corresponding formalin-fixed paraffin-embedded (FFPE) tissue blocks, blood specimens (1 mL aliquots of buffy coats, plasma/serum, and viable lymphocytes), genomic DNA, urine, and saliva. In addition, BRCF has established an electronic inventory system utilizing barcoding for sample identification, inventory tracking, and location (freezer number, shelf number, container number, and cell location) and annotating samples with basic demographic and clinical information. There is also a modular web-based software tool designed for BRCF to support operations that optimize the process of requesting and billing for biorepository services.

**Figure 2.**
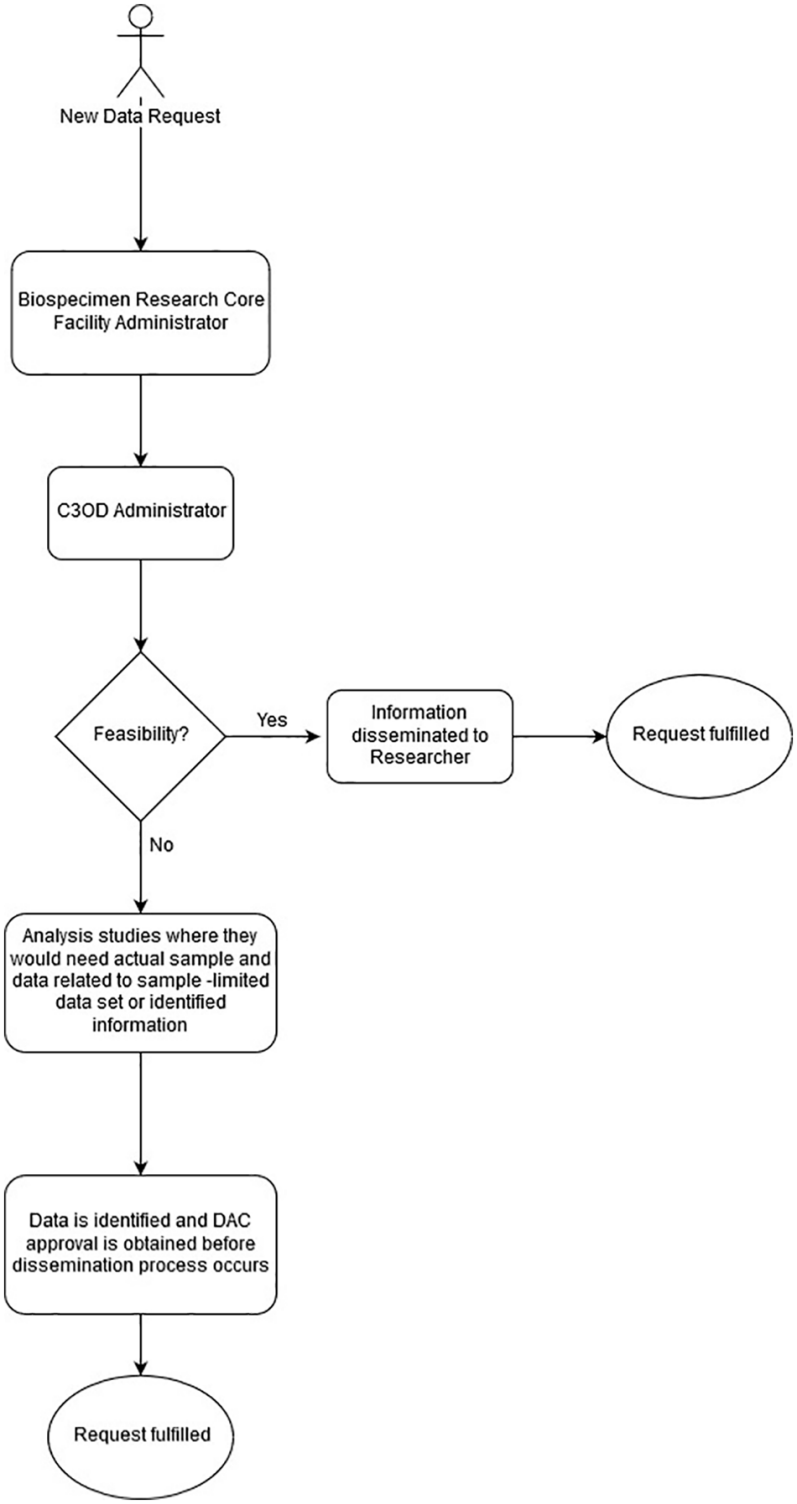
Biospecimen clinical annotation workflow. C3OD indicates Curated Cancer Clinical Outcomes Database; DAC, Data Access Committee.

The ability to link each specimen with data from C3OD enhances the value of each sample and reduces the amount of time needed to identify patients that match the researcher’s request criteria. To fulfill requests, the BRCF submits a project review to the DAC. The DAC will review the project and confirm use of identified data from C3OD will be used only for identifying patients as needed for the request and all appropriate IRB approvals from BRCF are received. Data Access Committee approves or disapproves the use of C3OD for the request. If approved, specimen identification can proceed. The BRCF data administrator interacts with C3OD to access clinical information. After generating a list of patients that match specified criteria, the BRCF data administrator determines whether the BRCF possesses the requested specimens, and C3OD specifies the counts of sample available for the requested sample type. The BRCF administrator also performs a cross verification with electronic health records (EHR) to confirm the list of patients—this is a spot check for a second verification, and the BRCF delivers requested samples redacted of protected health information to the researcher. An overview of this process is shown in Figure 2. Most requests are for the actual sample, not identified patient information, thus this process minimizes the number of people who need access to identifiable information. Under C3OD, EHR data is refreshed as needed and as often as daily, whereas Tumor registry data is refreshed once a month and biospecimen inventory information is updated once a week.

## Results

It is quite easy for the Informatics team to communicate with data managers as they are dealing with 3 to 4 managers at any given time who are fulfilling the BRCF requests. This also helps the informatics team to quickly update or tweak the data structure during the course of system updates that may occur at the source system. C3OD allows a more narrowed search of clinical criteria before moving to the final review of patient eligibility. Once a list of patients with basic tumor characteristics is created and before releasing samples to a researcher, the BRCF data managers will review additional information within the BRCF inventory system to correlate the collection date and EHR with final eligibility provided by the researcher, including but not limited to treatment status and disease status at the time of collection. C3OD has significantly reduced redundancy in the annotation of clinical data linked to biospecimen samples. Improving annotation has been a topic of attraction, which adds value to the samples that have been collected over a period of time. To utilize these samples efficiently, BRCF had to corroborate sample information with C3OD data elements. Prior to C3OD, manual clinical annotation by the BRCF data managers included disease diagnosis, staging, survival status, and other key data elements related to the patient, but more detailed information related to treatment and basic medical history is frequently needed. With C3OD, time to extract and annotate this information for each registered patient is essentially automated and allows access to details not previously annotated due to manual hours needed for each patient data extraction. In 2018, 10 of 51 projects (20%) submitted to the BRCF utilized C3OD in some fashion to complete the request. Some of the most common requests are described below. Since inception of C3OD, BRCF has utilized C3OD to complete approximately 25% of the data request that initiated through BRCF.

### Request 1

In the first of the 3 C3OD use-cases discussed in this report, investigators were interested in identifying ovarian cancer patients with high-grade serous disease with available FFPE tumor tissue and plasma samples banked within the BRCF.

A C3OD query using tumor registry data identified 504 patients (Morphology-Histology & Behavior ICD-O-3: [“8441” OR “8460” OR “8461”] and Primary Site: [“C569” OR “C482” OR “C481” OR “C570”]). From those patients, 193 matched with records in the BRCF specimen registry. Utilizing only our BRCF inventory system, we identified 328 ovarian cancer patients with available plasma samples. In the absence of C3OD, each patient’s EHR record would need to be reviewed to confirm the final histological diagnosis (high grade serous). C3OD enabled us to focus on the 193 and quickly narrow that down to those with annotated data available from the tumor registry before pulling FFPE for review. Electronic health records review to confirm qualification for the request requires approximately 10 minutes per patient. Thus, narrowing the available cohort of samples from 328 to 193 equates to over 22 hours of time saved.

### Request 2

The second use-case involved the identification of patients with a rare diagnosis: inverted papilloma, sinonasal squamous cell carcinoma, sinonasal squamous cell carcinoma from inverted papilloma, or sinonasal undifferentiated carcinoma. A C3OD query from the tumor registry data identified 253 with malignancies in the primary sites discussed in person with the researcher (ICD-O-3: C30.0, C31.0, C31.1, C31.2, C31.3, C31.9, C12.9). From those patients, 42 had matched records in the BRCF inventory system, and of those, 6 had frozen tissue available in the biorepository. C3OD enabled the BRCF data manager to quickly confirm the availability of a specific subset of tissue for the researcher.

### Request 3

The final use-case involved the identification of at least 50 frozen plasma samples for benign colorectal conditions. The BRCF patient registration focuses on patients with malignant disease; thus, annotation within the BRCF inventory system of non-malignancy is limited. A C3OD query of EHR data for 89 different ICD diagnosis codes related to benign colorectal conditions resulted in 13 699 unique patients. From those patients, 492 matched with records in the BRCF specimen registry with frozen plasma available. The BRCF provided the customer with 67 samples to meet the needs of the study.

As demonstrated in Table 1, we see that significant time and cost saving are utilizing a data-warehouse such as C3OD which is populated and curated to house quality data.

**Table 1.** Time and cost saving for BRCF team utilizing C3OD.

	Time		Cost (salaried time, $20/hr)	
	Before C3OD	After C3OD	Before C3OD	After C3OD
Request 1	54.67 hours	32.17 hours	US$1093.40	US$643.40
Request 2	Not possible	Minimal—<1 hour	Unknown	<US$20
Request 3	Not possible	11.17 hours	Unknown	US$224

Abbreviations: BRCF, Biospecimen Repository Core Facility; C3OD, Curated Cancer Clinical Outcomes Database.

## Discussion

C3OD improves efficiency and reduces the cost of fulfilling complex specimen requests within the BRCF. Without C3OD, the BRCF staff would be limited to only diagnosis information provided at the time of collection and limited historical patient information. Furthermore, each patient’s EHR would have to be reviewed to determine whether they meet the study criteria. C3OD quickly creates a list of patients that match many specific criteria and allows the BRCF staff to narrow down the number of records to review. Once a list of patients is created using C3OD, estimated time to confirm final qualifications with information in EHR is 5 to 10 minutes per patient. Our working group continues refinement of C3OD to improve the specificity of queries. Standardization in collecting data and extracting discrete data from unstructured documents are key areas of C3OD improvement in the future.

## Supplemental Material

suppl_file_xyz277344ddbed66 – Supplemental material for Optimizing Retrieval of Biospecimens Using the Curated Cancer Clinical Outcomes Database (C3OD) Click here for additional data file. Supplemental material, suppl_file_xyz277344ddbed66 for Optimizing Retrieval of Biospecimens Using the Curated Cancer Clinical Outcomes Database (C3OD) by Dinesh Pal Mudaranthakam, Elena Shergina, Michele Park, Jeffrey Thompson, David Streeter, Jinxiang Hu, Jo Wick, Byron Gajewski, Devin C Koestler, Andrew K Godwin, Roy A Jensen and Matthew S Mayo in Cancer Informatics

## References

[1] Jacobson RS Becich MJ Bollag RJ, et al A federated network for translational cancer research using clinical data and biospecimens. Cancer Res. 2015;75:5194-5201. 26670560 10.1158/0008-5472.CAN-15-1973 PMC4683415

[2] Liu A. Developing an institutional cancer biorepository for personalized medicine. Clin Biochem. 2014;47:293-299. 24373923 10.1016/j.clinbiochem.2013.12.015

[3] Campbell LD Astrin JJ DeSouza Y, et al The 2018 revision of the ISBER best practices: summary of changes and the editorial team’s development process. Biopreserv Biobank. 2018;16:3-6. 29393664 10.1089/bio.2018.0001 PMC5846567

[4] Office of Biorepositories and Biospecimen Research, NCI, NIH, U.S. Department of Health and Human Services. NCI Best Practices for Biospecimen Resources. https://biospecimens.cancer.gov/bestpractices/2016-NCIBestPractices.pdf. Accessed November 5, 2018.

[5] Kelly SM Wiehagen LT Schumacher PE Dhir R. Methods to improve sustainability of a large academic biorepository. Biopreserv Biobank. 2017;15:31-36. 27860489 10.1089/bio.2016.0076 PMC5326922

[6] Roden DM Pulley JM Basford MA, et al Development of a large-scale de-identified DNA biobank to enable personalized medicine. Clin Pharmacol Ther. 2008;84:362-369. 18500243 10.1038/clpt.2008.89 PMC3763939

[7] Bourgeois FT Avillach P Kong SW, et al Development of the precision link biobank at Boston Children’s Hospital: challenges and opportunities. J Pers Med. 2017;7:E21. 10.3390/jpm7040021 PMC5748633 29244735

[8] Foran DJ Chen W Chu H, et al Roadmap to a comprehensive clinical data warehouse for precision medicine applications in oncology. Cancer Inform. 2017;16:1176935117694349. 10.1177/1176935117694349 PMC5392017 28469389

[9] Mudaranthakam DP Thompson J Hu J, et al A Curated Cancer Clinical Outcomes Database (C3OD) for accelerating patient recruitment in cancer clinical trials. JAMIA Open. 2018;1:166-171. 30474074 10.1093/jamiaopen/ooy023 PMC6241508

