# Supplementary material for: Optimizing Retrieval of Biospecimens Using the Curated Cancer Clinical Outcomes Database (C3OD)
Source: Cancer Inform. 2019 Nov 18;18:1176935119886831. doi: 10.1177/1176935119886831 (PMC6864036; doi:10.1177/1176935119886831)
Supplement: suppl_file_xyz277344ddbed66 – Supplemental material for Optimizing Retrieval of Biospecimens Using the Curated Cancer Clinical Outcomes Database (C3OD) [file suppl_file_xyz277344ddbed66.pdf]

| Item Name | Original Order | Section | NAACCR field name | User Friendly field name |
|---|---|---|---|---|
| Histology ICD-O-3 | 270 | Cancer Identification | Histologic Type ICD-O-3 | |
| Behavior ICD-O-3 | 214 | Cancer Identification | Behavior Code ICD-O-3 | |
| Date Conclusive DX | 272 | Cancer Identification | Date Conclusive DX | Date of Conclusive Diagnosis |
| Date of Diagnosis | 70 | Cancer Identification | Date of Diagnosis | |
| Diagnostic Confirmation | 246 | Cancer Identification | Diagnostic Confirmation | |
| Grade | 115 | Cancer Identification | Grade | |
| Histology ICD-O-2 | 173 | Cancer Identification | Histology (92-00) ICD-O-2 | |
| Primary Site | 64 | Cancer Identification | Primary Site | |
| | 23 | Demographics | Medical Record Number | |
| Age | 43 | Demographic | Age at Diagnosis | |
| County | 286 | Demographic | County at DX | |
| Date of Birth | 24 | Demographic | Date of Birth | |
| Marital Status | 283 | Demographic | Marital Status at DX | |
| Place of Death- Country | 168 | Demographic | Place of Death--Country | |
| Place of Death- State | 22 | Demographic | Place of Death--State | |
| Race 1 | 280 | Demographic | Race 1 | |
| Race 2 | 289 | Demographic | Race 2 | |
| Sex | 281 | Demographic | Sex | |
| Spanish/Hispanic Origin | 130 | Demographic | Spanish/Hispanic Origin | |
| State | 207 | Demographic | Addr at DX--State | |
| Zip | 57 | Demographic | Addr at DX--Postal Code | Zip Code at Diagnosis |
| Cancer Status | 267 | Follow-up/Recurrence/Death | Cancer Status | |
| Cause of Death | 110 | Follow-up/Recurrence/Death | Cause of Death | |
| Follow-Up Source | 113 | Follow-up/Recurrence/Death | Follow-Up Source | |
| Place of Death | 97 | Follow-up/Recurrence/Death | Place of Death | |
| Recurrence Date--1st | 150 | Follow-up/Recurrence/Death | Recurrence Date--1st | |
| Recurrence Type--1st | 7 | Follow-up/Recurrence/Death | Recurrence Type--1st | |
| Vital Status | 135 | Follow-up/Recurrence/Death | Vital Status | |
| BRM/Immunotherapy Details | 256 | Hospital-Specific | RX Hosp--BRM | |
| Class of Case | 277 | Hospital-Specific | Class of Case | |
| Date of 1st Visit | 269 | Hospital-Specific | Date of 1st Contact | |
| Inpatient Status | 209 | Hospital-Specific | Inpatient Status | |
| RX Hosp--Chemo | 284 | Hospital-Specific | RX Hosp--Chemo | |
| RX Hosp--Surg App 2010 | 201 | Hospital-Specific | RX Hosp--Surg App 2010 | |
| RX Hosp--Surg Oth 98-02 | 200 | Hospital-Specific | RX Hosp--Surg Oth 98-02 | |
| Comorbid/Complication 1 | 180 | Stage/Prognostic Factors | Comorbid/Complication 1 | |
| Comorbid/Complication 10 | 51 | Stage/Prognostic Factors | Comorbid/Complication 10 | |
| Comorbid/Complication 3 | 197 | Stage/Prognostic Factors | Comorbid/Complication 3 | |
| Comorbid/Complication 5 | 13 | Stage/Prognostic Factors | Comorbid/Complication 5 | |
| Comorbid/Complication 6 | 139 | Stage/Prognostic Factors | Comorbid/Complication 6 | |

| | | | | |
|---|---|---|---|---|
| Comorbid/Complication 7 | 158 | Stage/Prognostic Factors | Comorbid/Complication 7 | |
| Comorbid/Complication 8 | 162 | Stage/Prognostic Factors | Comorbid/Complication 8 | |
| Comorbid/Complication 9 | 181 | Stage/Prognostic Factors | Comorbid/Complication 9 | |
| CS Extension | 205 | Stage/Prognostic Factors | CS Extension | |
| CS Lymph Nodes Eval | 55 | Stage/Prognostic Factors | CS Lymph Nodes Eval | |
| CS Mets at DX | 6 | Stage/Prognostic Factors | CS Mets at DX | |
| CS Mets at Dx-Brain | 123 | Stage/Prognostic Factors | CS Mets at Dx-Brain | |
| CS Mets at Dx-Liver | 36 | Stage/Prognostic Factors | CS Mets at Dx-Liver | |
| CS Mets at Dx-Lung | 128 | Stage/Prognostic Factors | CS Mets at Dx-Lung | |
| CS Mets Eval | 249 | Stage/Prognostic Factors | CS Mets Eval | |
| CS Site-Specific Factor 2 | 105 | Stage/Prognostic Factors | CS Site-Specific Factor 2 | |
| CS Site-Specific Factor 3 | 69 | Stage/Prognostic Factors | CS Site-Specific Factor 3 | |
| CS Site-Specific Factor 5 | 4 | Stage/Prognostic Factors | CS Site-Specific Factor 5 | |
| CS Site-Specific Factor 7 | 202 | Stage/Prognostic Factors | CS Site-Specific Factor 7 | |
| CS Site-Specific Factor 9 | 219 | Stage/Prognostic Factors | CS Site-Specific Factor 9 | |
| CS Site-Specific Factor11 | 94 | Stage/Prognostic Factors | CS Site-Specific Factor11 | |
| CS Site-Specific Factor12 | 91 | Stage/Prognostic Factors | CS Site-Specific Factor12 | |
| CS Site-Specific Factor13 | 96 | Stage/Prognostic Factors | CS Site-Specific Factor13 | |
| CS Site-Specific Factor14 | 81 | Stage/Prognostic Factors | CS Site-Specific Factor14 | |
| CS Site-Specific Factor15 | 53 | Stage/Prognostic Factors | CS Site-Specific Factor15 | |
| CS Site-Specific Factor16 | 264 | Stage/Prognostic Factors | CS Site-Specific Factor16 | |
| CS Site-Specific Factor17 | 74 | Stage/Prognostic Factors | CS Site-Specific Factor17 | |
| CS Site-Specific Factor18 | 41 | Stage/Prognostic Factors | CS Site-Specific Factor18 | |
| CS Site-Specific Factor19 | 252 | Stage/Prognostic Factors | CS Site-Specific Factor19 | |
| CS Site-Specific Factor23 | 28 | Stage/Prognostic Factors | CS Site-Specific Factor23 | |
| CS Tumor Size/Ext Eval | 73 | Stage/Prognostic Factors | CS Tumor Size/Ext Eval | |
| Derived AJCC-6 M Descript | 17 | Stage/Prognostic Factors | Derived AJCC-6 M Descript | |
| Derived AJCC-6 N Descript | 90 | Stage/Prognostic Factors | Derived AJCC-6 N Descript | |
| Derived AJCC-6 T Descript | 260 | Stage/Prognostic Factors | Derived AJCC-6 T Descript | |
| Lmphovascular Invasion/ Vascul... | 1 | Stage/Prognostic Factors | Lymph-vascular Invasion | |
| Number of LN Examined | 282 | Stage/Prognostic Factors | Number of LN Examined Regional Nodes Examined | |
| Number of Positive LN/ Lymph N... | 137 | Stage/Prognostic Factors | Number of Positive LN/ Lymph N.. Regional Nodes Positive | |
| Pediatric Staged By | 172 | Stage/Prognostic Factors | Pediatric Staged By | |
| Pediatric Staging System | 196 | Stage/Prognostic Factors | Pediatric Staging System | |
| TNM Path T | 145 | Stage/Prognostic Factors | TNM Path T | |
| Comorbid/Complication 2 | 10 | Stage/Prognostic Factors | Comorbid/Complication 2 | |
| Comorbid/Complication 4 | 62 | Stage/Prognostic Factors | Comorbid/Complication 4 | |
| CS Lymph Nodes | 126 | Stage/Prognostic Factors | CS Lymph Nodes | |
| CS Mets at Dx-Bone | 167 | Stage/Prognostic Factors | CS Mets at Dx-Bone | |
| CS Site-Specific Factor 1 | 49 | Stage/Prognostic Factors | CS Site-Specific Factor 1 | |
| CS Site-Specific Factor 4 | 101 | Stage/Prognostic Factors | CS Site-Specific Factor 4 | |

| | | | | |
|---|---|---|---|---|
| CS Site-Specific Factor 6 | 191 | Stage/Prognostic Factors | CS Site-Specific Factor 6 | |
| CS Site-Specific Factor 8 | 189 | Stage/Prognostic Factors | CS Site-Specific Factor 8 | |
| CS Site-Specific Factor10 | 93 | Stage/Prognostic Factors | CS Site-Specific Factor10 | |
| CS Site-Specific Factor20 | 212 | Stage/Prognostic Factors | CS Site-Specific Factor20 | |
| CS Site-Specific Factor21 | 153 | Stage/Prognostic Factors | CS Site-Specific Factor21 | |
| CS Site-Specific Factor22 | 86 | Stage/Prognostic Factors | CS Site-Specific Factor22 | |
| CS Site-Specific Factor24 | 117 | Stage/Prognostic Factors | CS Site-Specific Factor24 | |
| CS Site-Specific Factor25 | 30 | Stage/Prognostic Factors | CS Site-Specific Factor25 | |
| CS Tumor Size | 76 | Stage/Prognostic Factors | CS Tumor Size | |
| Derived AJCC-6 M | 16 | Stage/Prognostic Factors | Derived AJCC-6 M | |
| Derived AJCC-6 N | 61 | Stage/Prognostic Factors | Derived AJCC-6 N | |
| Derived AJCC-6 Stage Grp | 268 | Stage/Prognostic Factors | Derived AJCC-6 Stage Grp | |
| Derived AJCC-6 T | 182 | Stage/Prognostic Factors | Derived AJCC-6 T | |
| Derived AJCC-7 M | 211 | Stage/Prognostic Factors | Derived AJCC-7 M | |
| Derived AJCC-7 M Descript | 258 | Stage/Prognostic Factors | Derived AJCC-7 M Descript | |
| Derived AJCC-7 N | 174 | Stage/Prognostic Factors | Derived AJCC-7 N | |
| Derived AJCC-7 N Descript | 154 | Stage/Prognostic Factors | Derived AJCC-7 N Descript | |
| Derived AJCC-7 Stage Grp | 78 | Stage/Prognostic Factors | Derived AJCC-7 Stage Grp | |
| Derived AJCC-7 T | 229 | Stage/Prognostic Factors | Derived AJCC-7 T | |
| Derived AJCC-7 T Descript | 179 | Stage/Prognostic Factors | Derived AJCC-7 T Descript | |
| Extent of Disease | 50 | Stage/Prognostic Factors | Extent of Disease 10-Dig | |
| Pediatric Stage | 253 | Stage/Prognostic Factors | Pediatric Stage | |
| SEER Summary Stage 1977 | 279 | Stage/Prognostic Factors | SEER Summary Stage 1977 | |
| SEER Summary Stage 2000 | 215 | Stage/Prognostic Factors | SEER Summary Stage 2000 | |
| TNM Clin Descriptor | 32 | Stage/Prognostic Factors | TNM Clin Descriptor | |
| TNM Clin M | 20 | Stage/Prognostic Factors | TNM Clin M | |
| TNM Clin N | 242 | Stage/Prognostic Factors | TNM Clin N | |
| TNM Clin Stage Group | 127 | Stage/Prognostic Factors | TNM Clin Stage Group | |
| TNM Clin Staged By | 193 | Stage/Prognostic Factors | TNM Clin Staged By | |
| TNM Clin T | 147 | Stage/Prognostic Factors | TNM Clin T | |
| TNM Edition Number | 37 | Stage/Prognostic Factors | TNM Edition Number | |
| TNM Path Descriptor | 63 | Stage/Prognostic Factors | TNM Path Descriptor | |
| TNM Path M | 108 | Stage/Prognostic Factors | TNM Path M | |
| TNM Path N | 271 | Stage/Prognostic Factors | TNM Path N | |
| TNM Path Stage Group | 60 | Stage/Prognostic Factors | TNM Path Stage Group | |
| TNM Path Staged By | 143 | Stage/Prognostic Factors | TNM Path Staged By | |
| Date 1st Crs RX CoC | 67 | Treatment-1st Course | Date 1st Crs RX CoC | |
| Rad--Boost Dose cGy | 58 | Treatment-1st Course | Rad--Boost Dose cGy | |
| Rad--Boost RX Modality | 204 | Treatment-1st Course | Rad--Boost RX Modality | |
| Rad--Location of RX | 251 | Treatment-1st Course | Rad--Location of RX | |
| Rad--No of Treatment Vol | 52 | Treatment-1st Course | Rad--No of Treatment Vol | |

| | | | | |
|---|---|---|---|---|
| Readm Same Hosp 30 Days | 176 | Treatment-1st Course | Readm Same Hosp 30 Days | |
| Reason for No Radiation | 109 | Treatment-1st Course | Reason for No Radiation | |
| Reason for No Surgery | 144 | Treatment-1st Course | Reason for No Surgery | |
| RX Summ--Chemo | 234 | Treatment-1st Course | RX Summ--Chemo | |
| RX Summ--Hormone | 68 | Treatment-1st Course | RX Summ--Hormone | |
| RX Summ--Other | 259 | Treatment-1st Course | RX Summ--Other | |
| RX Summ--Palliative Proc | 210 | Treatment-1st Course | RX Summ--Palliative Proc | |
| RX Summ--Rad to CNS | 185 | Treatment-1st Course | RX Summ--Rad to CNS | |
| RX Summ--Radiation | 14 | Treatment-1st Course | RX Summ--Radiation | |
| RX Summ--Reconstruct 1st | 39 | Treatment-1st Course | RX Summ--Reconstruct 1st | |
| RX Summ--Reg LN Examined | 89 | Treatment-1st Course | RX Summ--Reg LN Examined | |
| RX Summ--Scope Reg LN Sur | 134 | Treatment-1st Course | RX Summ--Scope Reg LN Sur | |
| RX Summ--Surg Prim Site | 38 | Treatment-1st Course | RX Summ--Surg Prim Site | |
| RX Summ--Surgery Type | 107 | Treatment-1st Course | RX Summ--Surgery Type | |
| RX Summ--Surgical Approch | 206 | Treatment-1st Course | RX Summ--Surgical Approch | |
| RX Summ--Systemic/Sur Seq | 254 | Treatment-1st Course | RX Summ--Systemic/Sur Seq | |
| RX Summ--Transplnt/Endocr | 124 | Treatment-1st Course | RX Summ--Transplnt/Endocr | |
| RX Summ--Treatment Status | 226 | Treatment-1st Course | RX Summ--Treatment Status | |
| Date Initial RX SEER | 26 | Treatment-1st Course | Date Initial RX SEER | |
| Rad--Regional Dose: cGy | 71 | Treatment-1st Course | Rad--Regional Dose: cGy | |
| Rad--Regional RX Modality | 83 | Treatment-1st Course | Rad--Regional RX Modality | |
| Rad--Treatment Volume | 85 | Treatment-1st Course | Rad--Treatment Volume | |
| RX Coding System--Current | 261 | Treatment-1st Course | RX Coding System--Current | |
| RX Date BRM | 47 | Treatment-1st Course | RX Date BRM | |
| RX Date Chemo | 230 | Treatment-1st Course | RX Date Chemo | |
| RX Date DX/Stg Proc | 103 | Treatment-1st Course | RX Date DX/Stg Proc | |
| RX Date Hormone | 112 | Treatment-1st Course | RX Date Hormone | |
| RX Date Other | 18 | Treatment-1st Course | RX Date Other | |
| RX Date Rad Ended | 141 | Treatment-1st Course | RX Date Rad Ended | |
| RX Date Radiation | 131 | Treatment-1st Course | RX Date Radiation | |
| RX Date Surgery | 88 | Treatment-1st Course | RX Date Surgery | |
| RX Date Systemic | 27 | Treatment-1st Course | RX Date Systemic | |
| RX Summ--BRM | 265 | Treatment-1st Course | RX Summ--BRM | |
| RX Summ--DX/Stg Proc | 77 | Treatment-1st Course | RX Summ--DX/Stg Proc | |
| RX Summ--Surg Oth Reg/Dis | 42 | Treatment-1st Course | RX Summ--Surg Oth Reg/Dis | |
| RX Summ--Surg/Rad Seq | 187 | Treatment-1st Course | RX Summ--Surg/Rad Seq | |
| RX Summ--Surgical Margins | 114 | Treatment-1st Course | RX Summ--Surgical Margins | |
